# Performance and Usability of Various Robotic Arm Control Modes from Human Force Signals

**DOI:** 10.3389/fnbot.2017.00055

**Published:** 2017-10-25

**Authors:** Sébastien Mick, Daniel Cattaert, Florent Paclet, Pierre-Yves Oudeyer, Aymar de Rugy

**Affiliations:** ^1^Flowers Team, INRIA Bordeaux Sud-Ouest, Talence, France; ^2^Hybrid Team, Institut de Neurosciences Cognitives et Intégratives d’Aquitaine, CNRS UMR 5287, Univ. Bordeaux, Bordeaux, France; ^3^Centre for Sensorimotor Performance, School of Human Movement and Nutrition Sciences, University of Queensland, Brisbane, QLD, Australia

**Keywords:** neuroprosthesis, arm prosthesis, robotic arm, real-time control, control mode, usability testing

## Abstract

Elaborating an efficient and usable mapping between input commands and output movements is still a key challenge for the design of robotic arm prostheses. In order to address this issue, we present and compare three different control modes, by assessing them in terms of performance as well as general usability. Using an isometric force transducer as the command device, these modes convert the force input signal into either a position or a velocity vector, whose magnitude is linearly or quadratically related to force input magnitude. With the robotic arm from the open source 3D-printed Poppy Humanoid platform simulating a mobile prosthesis, an experiment was carried out with eighteen able-bodied subjects performing a 3-D target-reaching task using each of the three modes. The subjects were given questionnaires to evaluate the quality of their experience with each mode, providing an assessment of their global usability in the context of the task. According to performance metrics and questionnaire results, velocity control modes were found to perform better than position control mode in terms of accuracy and quality of control as well as user satisfaction and comfort. Subjects also seemed to favor quadratic velocity control over linear (proportional) velocity control, even if these two modes did not clearly distinguish from one another when it comes to performance and usability assessment. These results highlight the need to take into account user experience as one of the key criteria for the design of control modes intended to operate limb prostheses.

## 1 Introduction

In the field of robotic prostheses, development in neuroscience, bioimagery, and physiological monitoring led to the introduction of multiple techniques aiming at providing a disabled subject with the control of the actuators of a prosthesis. Whether the disability is congenital (limb agenesia) or of traumatic origin (amputation), these systems make use of the subject’s remaining or unaltered abilities, allowing the subject to generate biological signals that can be detected and interpreted as motor commands. Unlike body-powered prostheses, which employ purely mechanical systems to convert limited residual motor ability into more complex movements and gestures, robotic prostheses rely on the measurement and processing of physiological activity to operate the prosthesis actuators.

Various methods and tools developed in the field of such prostheses propose different approaches for interpreting physiological signals to drive a limb prosthesis. From recent works on sensorimotor systems, several techniques employ brain–computer interfaces (BCI) recording neuronal activity, such as intra-cortical electrode matrices located in motor areas (Kim et al., 2008; Gilja et al., 2012; Sussillo et al., 2012; Golub et al., 2014), or surface electroencephalography (EEG) devices (Li et al., 2010). However, due to their invasiveness and/or low acceptability, most of these techniques are currently limited to small-scale clinical use or experimental applications and have yet to be implemented on commercial systems.

On the other hand, myoelectric systems rely on electromyographic (EMG) signals measured with surface electrodes located on muscles remaining on or near the disabled limb. In order to process these signals and extract motor or neural information from them, several techniques and approaches have been developed (Oskoei and Hu, 2007) and are used in commercial or clinical context. Contrary to neural interfaces, EMG-based interfaces do not require prior surgical intervention, which makes their application easier for prosthesis control. Additionally, a survey on people suffering from upper-limb loss showed that current and potential upper-limb prostheses users were generally more interested in myoelectric devices than in other neuroprosthesis control techniques (Engdahl et al., 2015). Due to these decisive advantages, surface EMG signals are the only input type to be extensively employed in commercial upper-limb prosthesis control (Farina et al., 2014). Regarding recent works in the literature, EMG signals have been used as input signals for decoding wrist joint movements (Hahne et al., 2015), as well as limb-produced forces in a static context (no joint motion allowed) (de Rugy et al., 2012b; Berger et al., 2013).

In the present work, the command interface is a force sensor, measuring linear efforts produced by the hand of a healthy human subject. Although these force measurements act here as the primary command input from the subjects, they can be viewed as an intermediary signal throughout the processing of muscular activities into output motion. Indeed, de Rugy et al. (2012b) showed that in an isometric context, wrist forces are rather easy to reconstruct from myoelectric signals, through basic linear regression. In the case of myoelectric prostheses, the isometric constraint appears to be relevant: a disabled patient’s residual motor ability would produce muscular activity without generating any joint motion of the disabled limb. Furthermore, Berger et al. (2013) and de Rugy et al. (2012a) demonstrated that the pattern of muscle coordination used to produce forces during arm movements was not quite flexible, making it harder for a subject to elaborate or adapt to novel muscle coordination patterns when generating efforts with the arm. As a result, addressing the control by force signals represents a relevant step in the development of myoelectric arm prostheses.

In order to operate a mobile device, such as a motor-driven prosthesis or a cursor on a screen, the previously mentioned systems employed methods that converted neuronal or muscular activity into kinematic information describing the intended motion from a spatio-temporal perspective. We call such methods *control modes*, as they represent different ways to process physiological input signals in order to control the prosthesis’s motion. Some of these control modes rely on the production of native arm movements as a basis for decoding the association between physiological activity and endpoint or joint angle trajectory, with techniques such as Kalman filters (Kim et al., 2008; Gilja et al., 2012; Golub et al., 2014), neural networks (Sussillo et al., 2012), or linear regression (Hahne et al., 2015). Such techniques propose a reconstruction approach, that is to say: investigate and emulate an existing relationship between physiological signals and genuine arm movements in order to reproduce similar movements.

On the other hand, a control mode can also operate as an abstract relationship between command signals and output movements, regardless of any native or natural reference. Of course, such ways for the user to put the device in motion are still intended to be relevant to the use cases, the expected users and the mechanical properties of the system. Similarly, video games which put the player in control of a movable character or item, as well as tele-operation devices, necessarily propose (and often impose) a control mode as a mapping between interface elements (buttons, joystick, mouse, touchscreen, etc.) and the kinematic behavior of the mobile item. Unlike limb prostheses, there may not exist any *natural* motion associated to the mobile item, so this mapping may be subjectively designed instead of being elaborated from genuine movements.

To this day, from the systems found in video games and telemanipulators as well as in the BCI and neuroprostheses fields, we can note that existing control modes rely on various types of kinematic quantities to drive the mobile device. The most common type is kinetic quantities, such as velocity, typically featured when the command interface is a keyboard, or an isometric device, i.e., which is operated through force or torque, such as a spring-mounted joystick. Another type is positional quantities (e.g., coordinates, angular position), typically featured when the command interface is an isotonic device, i.e., which is operated through movement or displacement, such as a Wii Remote™ or a computer mouse. In the same way as with video games and tele-operated devices, both of these kinematic types can be employed to drive a robotic prosthesis’s endpoint. Besides, several types of transfer functions converting the input signal into said quantity can be implemented and evaluated. For example, Casiez et al. (2008) compared constant Cursor-Display (CD) gain to non-linear speed-dependent CD for mouse-driven cursor position control, concluding that the latter performs slightly better, and Poupyrev et al. (1996) elaborated a biphasic transfer function combining constant gain and quadratic gain for 3-D hand-driven position control in a virtual reality environment, providing a greater reachable space than with constant gain, but still allowing for easy and intuitive manipulation at close range. These two works support the fact that non-linear transfer functions may perform better because of their versatility, by achieving good performance at both close and long range. Moreover, Kantowitz and Elvers (1988) addressed how different constant CD gain values interact with order of control (velocity or position), showing that high CD gain relatively draws more benefits with velocity control than with position control.

Designing a control mode requires to determine which kinematic types to use, as well as which transfer function to implement, depending on the nature of the command device and the input signals. For example, the results of Teather and MacKenzie (2014) favor position control over velocity control for tilt-driven cursor motion on a tablet screen; Kim et al. (1987) came to the same conclusion by carrying out a similar comparison employing a virtual telemanipulator, driven with a joystick as the command device, and Zhai (1995) advised to associate rate (velocity) control with isometric devices but position control with isotonic devices, for hand-held controllers with up to 6 degrees of freedom (DoF). In the case of BCI decoders, several works such as Gilja et al. (2012) and Kim et al. (2008) studied or commented the benefits of one kinematic quantity over another, the former providing neurophysiology-oriented arguments in favor of velocity control over position control, the latter demonstrating a better control of cursor movements based on speed direction than on speed magnitude. Similarly, Golub et al. (2014) addressed the improvement of a velocity control mode by enhancing movement direction over movement speed in the processing of intra-cortical activity into cursor motion. However, these works compare processes that use the same input (physiological activity) and the same reference motion (native limb movements) to investigate how different kinematics quantities from native movements can be decoded from the physiological input. No insight is provided regarding systems converting the same input into different kinematic quantities producing different motions, which may not correspond to native limb movements.

In the present work, we use a robotic arm endpoint as the mobile device. Three different control modes are designed, relying on the same type of command input from the force sensor, but converting it into different kinematic quantities describing the arm’s endpoint motion to generate. Consequently, based on the same input from the force sensor, each control mode would produce a different endpoint motion. No reconstruction is performed: our approach does not address the decoding of *existing* input–output relationships, and we elaborate instead the different control modes as original input–output relationships.

On the other hand, regardless of input and output quantities, most recent developments in prosthesis control focused on elaborating an accurate mapping between physiological activities and resulting movements, without addressing aspects related to user experience. Indeed, most validation criteria used to demonstrate an improvement provided by a novel method correspond to performance metrics in the context of a target-reaching task, e.g., movement time, path length, change in movement direction (Gilja et al., 2012; Sussillo et al., 2012; Hahne et al., 2015). We propose here to complement these performance metrics by assessing usability aspects for different control modes. As a property of a system, usability is the degree to which the system can provide a satisfactory interaction with its users. Studies of this property often concern digital interfaces displayed on a screen (Holzinger, 2005; Nielsen, 2005), but usability can also be assessed for other types of systems working with a human user (Logan et al., 1994; Buurke et al., 1999; Haak et al., 2007). As defined in International Organization for Standardization (1998), usability can be split up into three key aspects:

- effectiveness: to what extent the system allows the user to correctly perform a task
- efficiency: to what extent using the system requires effort from the user, e.g., learning, focus, time spent, fatigue.
- satisfaction: to what extent the user subjectively appreciates using the system.

Although this definition was elaborated for digital interface design, it does not integrate any condition regarding the nature of the system nor the user, and thus remains applicable to other systems. In the field of robotic prostheses, usability plays a crucial role: a poorly usable prosthesis may not sufficiently compensate the patient’s disability, possibly leading to rejection if the patient would rather use the deficient limb (Biddiss and Chau, 2007; Resnik, 2011). In this context, control modes can greatly influence user experience: this parameter determines the way the prosthesis is put in motion, which will be confronted to the patient’s expectations, needs, and limitations in terms of motricity. Regarding these aspects, the most common approach assumes that emulating native human movements ensures that the prosthesis behavior will be closest to a valid subject’s natural movements. To our knowledge, no work on neuroprostheses carries out a comparison of several decoders or control modes in terms of usability, and most existing works assume that the best decoding or controlling system is the one allowing for the best motor performance.

In order to address the influence of control modes on usability in the operation of a robotic arm prosthesis, we designed an experimental framework centered on a target-reaching task, and carried out tests with able-bodied subjects. The usability assessment relies on performance metrics on the one hand, and a post-experiment questionnaire on the other hand, in order to explore the multiple dimensions of the system’s usability rather than only focus on measurements evaluating skills and performance.

## 2 Materials and Methods

### 2.1 Participants

The study was conducted on a set of 18 able-bodied naive subjects (11 male), aged 20–51 (mean 27.7; SD 9.4), none of them suffering of any mental or motor disorder that could affect their ability to perform the task. The experiment duration ranged from approximately 30 to 45 min, and no subject reported fatigue at the end of the experiment.

This study was carried out in accordance with the recommendations of the local ethics committee (CPP Sud-Ouest et Outre Mer III, Ref DC2014/16), with written informed consent from all subjects. All subjects gave written informed consent in accordance with the Declaration of Helsinki. The protocol was approved by the local ethics committee.

### 2.2 Apparatus

**Figure 1:**
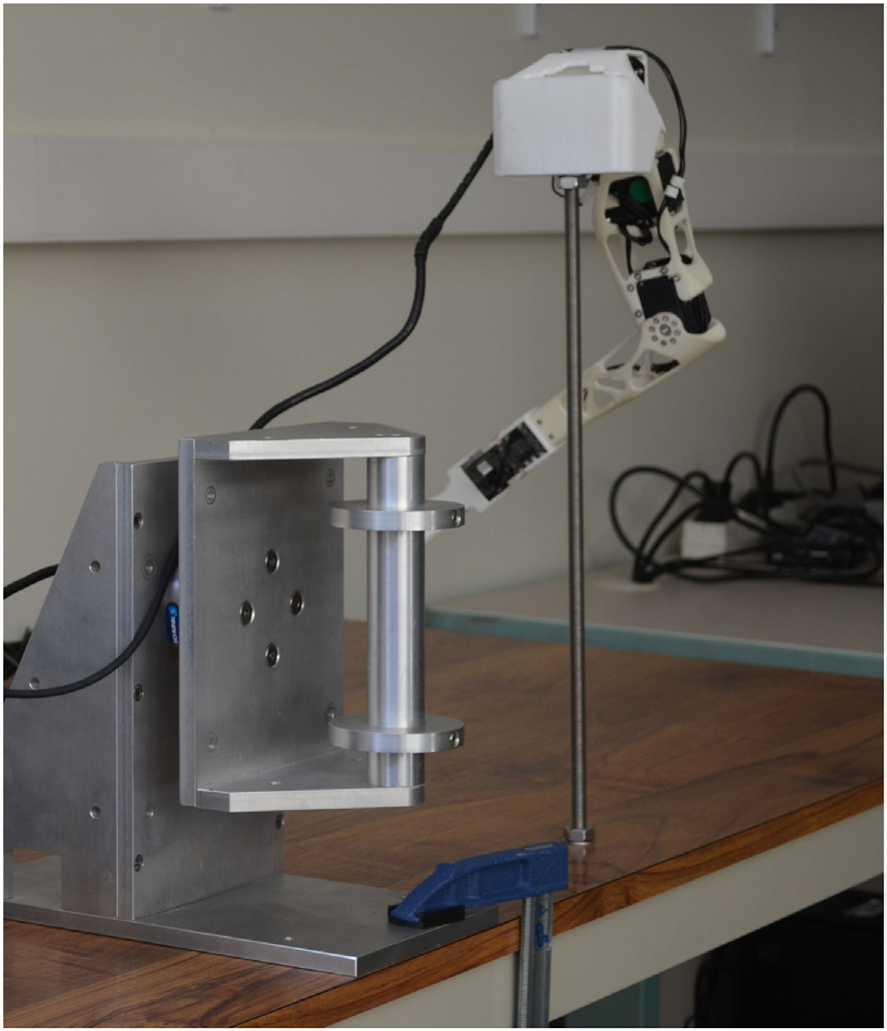
Experimental apparatus. The force sensor assembly is mounted next to the rod that holds the robot’s shoulder assembly.

A 6-axis force transducer (Delta F/T; ATI Industrial Automation) is mounted on a custom-made cylindrical handle on the edge of a table. The handle is 5-cm wide in diameter and 10-cm high, so that an adult human subject can easily and firmly grasp it with the hand (see Figure 1). Two wide stop parts are placed at each end of the handle, to prevent the subject’s hand from slipping along the handle. The sensor is used to measure a 3-dimensional vector representing the force applied on the handle. This vector acts as the command signal, representing the direction and intensity of the intended movement. The sensor is static: it does not have any movable part and the handle is at the same position and orientation at all times.

**Figure 2:**
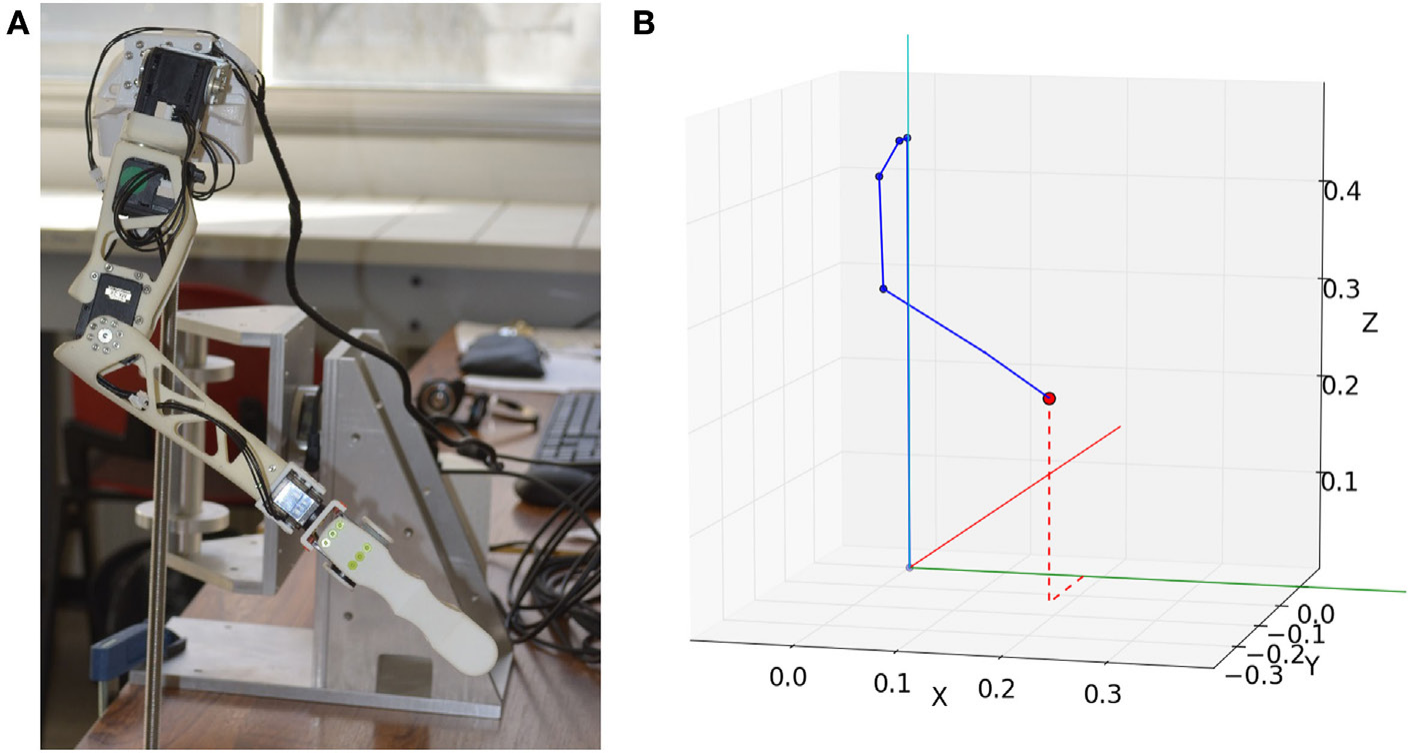
Robot arm and corresponding virtual kinematic chain. **(A)** Robot arm standing in its starting posture. **(B)** Corresponding virtual arm posture generated with inverse kinematics. The red dot represents the goal position. Each blue dot represents a joint. Each solid blue line represents a skeleton part.

A robotic arm acts as the physical controllable prosthesis (Figure 2A). This robotic device is a right arm taken from the Poppy Humanoid platform (Lapeyre et al., 2014), which aims at providing software and hardware architecture to easily build and operate robots, for science, education, and art. The mobile device employed in this work has roughly the size and proportions of a 3-year-old child’s limb, for a total arm length of approximately 50 cm and a total weight of about 400 g. It comprises four motors, corresponding to four rotating DoF, three of them located at shoulder level, the last one operating the elbow flexion-extension joint. The device is mounted on a vertical rod to which the shoulder of the robot is fixed. A short stick is mounted at wrist level to materialize the robot’s endpoint instead of the standard five-finger hand from the original Poppy Humanoid robot. The rod is placed on the table, next to the force transducer.

A mechanical model of the robotic arm was elaborated, describing from a geometrical point of view the relative position and orientation of each joint and skeleton part, from the robot’s base to its endpoint. The joints are ordered following a sequence that forms a kinematic chain. This model is then used to perform direct and inverse kinematics calculations with a dedicated software module (Manceron, 2015). By going through the kinematic chain step by step, direct kinematics determine the endpoint’s position in the 3-D space, based on the angular position of each motor. Reciprocally, inverse kinematics determine a set of motor angular positions that put the endpoint at a given goal in the 3-D space (Figure 2B).

### 2.3 Control *Modes*

We explored here two different orders of control for driving the robot’s endpoint in the 3-D space: position and velocity (also referred to as “rate”), which are the most commonly found in the literature on neuroprostheses (Kim et al., 2008; de Rugy et al., 2012b; Gilja et al., 2012; Sussillo et al., 2012; Golub et al., 2014; Hahne et al., 2015). Based on these orders of control, we defined three different methods to put the robot in motion depending on the force vector measured by the transducer. Each method converts this command vector into a goal value (position or velocity) describing the kinematic behavior of the arm’s endpoint in the 3-D space. These methods play the role of control modes, as they correspond to different ways of controlling the endpoint’s motion according to the intended movement described by the input command vector.

The endpoint is the only element of the robot arm that is taken into account by these control modes, as a mobile point within the 3-D operational space. The control modes do not work in the articular space (at motor angles level) and do not perform posture generation on the robot arm.

#### 2.3.1 Position Control Mode (Pos)

This control mode implements the most basic way to drive the endpoint’s position according to the command vector. It determines a goal position by proportionally converting the force vector into a displacement vector, in relation to a starting position. This method is closely similar to the force-driven cursor control system described in de Rugy et al. (2012a) and de Rugy et al. (2012b):

(1) $$\vec{OG}=k_{p}\cdot \vec{f}$$

where *O* is the starting position, *G* is the goal position, *k_p_* is a scalar gain and, $\vec{f}$ is the measured force vector.

In terms of magnitude, exerting a larger effort on the handle will bring the endpoint further from its starting position. In the case of a null effort, the endpoint will be brought and held at this starting position.

#### 2.3.2 Proportional Velocity Control Mode (Vel)

This mode implements the most basic way to drive the endpoint’s velocity according to the command vector. It determines a goal velocity as collinear to the force vector, pointing in the same way and of magnitude proportional to the force intensity:

(2) $$\vec{v_{G}}=k_{v}\cdot \vec{f}$$

where $\vec{v_{G}}$ is the goal velocity, *k_v_* is a scalar gain, and $\vec{f}$ is the measured force vector.

Exerting a larger effort on the handle will drag the endpoint faster in the direction of the measured vector, but not necessarily further, depending on its previous position. A null effort will make the robot stand still, so that the endpoint does not move.

#### 2.3.3 Quadratic Velocity Control Mode (Vel2)

This mode acts very similarly to the previous one, but introduces a nonlinear relationship in the force-to-speed conversion. It determines a goal velocity as collinear to the force vector, pointing in the same way and of magnitude proportional to the squared force intensity:

(3) $$\vec{v_{G}}=k_{v2}\cdot \left\|\vec{f}\right\|\cdot \vec{f}$$

where $\vec{v_{G}}$ is the goal velocity, *k_v_*_2_ is a scalar gain, and $\vec{f}$ is the measured force vector.

The quadratic relationship in magnitude allows the system to overcome one of the limits of the previous mode. Indeed, in the case of a proportional control, moving at high speed with reasonable effort requires a high scalar gain, which makes slow, accurate movements harder to achieve. A quadratic relationship allows this third mode to produce high speed movements for medium efforts without any massive loss of accuracy or fine control at low speeds. This mode was inspired by other examples of non-linear transfer function in mobile device control, such as the pointer acceleration technique, described by Casiez et al. (2008), and the Go–Go interaction technique proposed by Poupyrev et al. (1996).

#### 2.3.4 Motor Operation

Each of these control modes takes place within the motion generation process by converting the input force vector into a goal position or velocity. Then, inverse kinematics is employed to operate the robot’s motors so that the endpoint moves to comply best with this goal. The robotic device being still subject to its physical limits and properties, its motion does not perfectly fit the goal velocity or position. Besides, as a safety measure, motion restriction was implemented so that the robot is not put in dangerous postures with respect to body envelope and motor angular bounds.

From the perspective of the present work, the robot itself is just a tool, seen as a black box employing algorithms, electronic and mechanical components to put the endpoint in motion. With respect to the main concerns of the present work, the properties of the motors and algorithms employed to perform this control are not relevant, as long as they enable the endpoint’s movements to accurately conform to the goal position or velocity. The authors are aware that robot control and operation aspects have an influence on the collected data, but assume that these factors equally apply to the different control modes employed during the experiments, and thus do not affect how they compare to each other in terms of performance results. Please refer to Siciliano and Khatib (2016) for further insight on inverse kinematics and motor operation.

### 2.4 Task

The default posture of the robot places its endpoint at a starting position, at the center of a starting zone, a 2-cm radius sphere. The setup comprises five spherical targets (4 cm in diameter) distributed in the reachable space of the robot. Their centers are set at an equal distance of 19 cm from the starting point. The task is a center-out target-reaching task: the robot’s endpoint acts as a cursor and the goal is to move this cursor from this center to one of these targets. The task is successful if the endpoint remains within the target’s zone during at least 600 ms continuously. The subject is allowed a period of 25 s to complete this goal. Before the experiment begins, the task is described to the subject, which is then aware of the goal, success conditions and parameters of the experiment.

In order to display these targets to the subject, disks of same diameter (4 cm) are placed within the experimental setup, coinciding with the locations and general shape of the targets. These disks are made of foam padding and held in place with elastic rope, so that the assembly can absorb shocks without damaging the robotic arm in case of a collision during the task and still bring the targets back to their original locations. In that way, the subject can visually identify the target positions and dimension, while the robotic arm’s endpoint is still able to physically enter the target. Additionally, a short audio cue is played each time the endpoint gets into the spherical target zone. This feature provides supplementary feedback to the subject, allowing him/her to know if the endpoint is currently within the target zone, which is only partially marked out by the corresponding disk.

The choice of scalar gain values *k_p_, k_v_*, and *k_v_*_2_ is a key aspect of the apparatus configuration: these parameters determine the system’s sensitivity in relation to a force applied on the transducer. In our case, the dimensional heterogeneity of these three parameters is such that employing the same gain value with all three control modes would not guarantee their use in identical experimental conditions, as they fundamentally differ by the nature of their input/output relationship. These differences also clearly limit the possibility to choose values that could be considered strictly equivalent with respect to the task, although they should not be chosen independently from one another.

Besides, the choice of these gain values has a direct influence on the feasibility and difficulty of the task: an extreme value (e.g., too low to produce fast movements or too high to remain accurately controllable) could make the task impossible to perform successfully. However, several works such as Casiez et al. (2008) and Kim et al. (1987) compared performance achieved with different gain values and showed that, apart from such extreme values, gain variations had only minor effects on movement time. These results tend to indicate that within a “usable” interval, i.e., between the extreme values, scalar gains do not have a major influence on performance or task difficulty. Thus, we assume that, for a given control mode, employing a gain value picked out within this “usable” interval ensures that the task will be feasible for human subjects, without this choice significantly affecting the performance level.

From these findings, we chose the gain values by conducting a pilot testing with six naive, able-bodied subjects that were not involved in the main experiment. For each control mode, we tested various gain values, determined a “usable” interval and identified a medium value that allowed subjects to maneuver the robot arm with ease within the setup and successfully perform the task. Although the chosen gain values cannot be considered equivalent, the pilot testing guarantees that all control modes are correctly configured in respect to the task, allowing a comparison of their performance with certain fairness.

We detail here, for each mode, examples of force input needed to move the endpoint from its starting position to the target center, along a straight, 19-cm long line:

- Pos mode: 8.96 N
- Vel mode: 2.265 N applied during 4.61 s, or 5.225 N applied during 2 s
- Vel2 mode: 2.265 N applied during 4.61 s, or 3.44 N applied during 2 s.

2.265 N is the only input force magnitude at which both Vel and Vel2 modes generate the same speed. 4.61 s is the time needed to travel the 19-cm distance at that speed. For a quicker movement (e.g., 2 s movement time), the required force input with Vel2 mode (3.44 N) is lower than with Vel mode (5.225 N).

### 2.5 Protocol

During the experiment, the subject was seated in front of the sensor and placed so that the handle faces its right shoulder and the subject’s forearm remains horizontal when he/she grasps the handle with the right hand. The subject was asked to stand this way, with a straight back, during the experiment.

Before the experiment started, the experimenter presented the setup to the subject by describing that the sensor measures linear efforts along the 3 spatial directions, and that the robot is put in motion in the same direction as the measured effort. No further detail was provided to the subject regarding the way each control mode works. For each subject, the experiment was split into three series of twenty task trials. Each series was performed with one of the three available modes and comprised four trials for each of the five targets, for a total of sixty trials during the experiment. The order of these modes was shuffled so that overall, the learning and order effects are compensated among the population of subjects.

The target order was generated in a block-randomized fashion and common to all subjects and series. The label of the target to reach was revealed to the subject before the task begins. Then, the subject was given the control of the robot arm, the endpoint being placed at the center. When, under the subject’s control, the endpoint leaved the starting zone for the first time, the task timer started. The task finished at the end of the 25 s period or as soon as the endpoint was successfully maintained for at least 600 ms inside a target. We used the software PsychoPy (Peirce, 2008) to display target labels, handle the control loop, and time the different task events, e.g., leaving the starting zone for the first time, entering the target zone for the first time, staying the required time within the target zone.

### 2.6 Analysis

**Figure 3:**
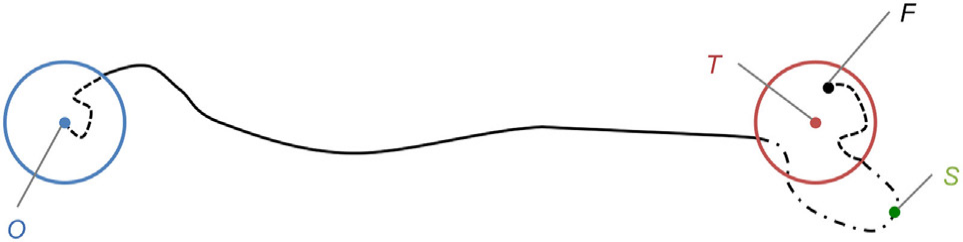
2-D representation of the task setup. *O* is the starting position, surrounded by the starting zone, in blue. *T* is the target center, surrounded by the target zone, in red. *F* is the final position, after a successful hold-inside phase. *S* is the reached point furthest from *O*. The path followed by the endpoint is drawn in dashed and solid lines.

In addition to the information of task success or failure, four measurements were performed during each trial (Figure 3):

1. *Acquisition Time (AT)*: time elapsed since the endpoint has exited the starting zone until it enters the target zone for the first time. It corresponds to the time elapsed on the path along the solid line. If the target zone was not entered at the end of the 25 s period, AT is set to 25 s. This commonly found metric (Gilja et al., 2012; Hahne et al., 2015) focuses on assessing overall movement speed, but also slightly addresses trajectory control, as reaching the target requires driving the endpoint toward the correct direction.
2. *Validation Time (VT)*: time elapsed since the endpoint has entered the target zone until the end of the 600 ms hold-inside period. It corresponds to the time elapsed on the path along the second dashed line, from the end of the solid line to *F*. If the endpoint was not successfully held inside the target zone for a sufficient time, VT is set to 25 s minus AT. Also referred to as “dial-in time” in Gilja et al. (2012) and Sussillo et al. (2012), this metric assesses mostly stability, i.e., to what extent the system allows fine control and low movement speed, in order to hold the endpoint on the target. It also addresses accuracy in the way that the holding phase requires a precise control of the endpoint position over time.
3. *Path Shortness (PS)*: ratio of the followed path length by the shortest path length, whether the target was reached or not. The total path length corresponds to the length of the dashed and solid lines from *O* to *F*. The shortest path length is the distance to the target (*OT*). Taken from Hahne et al. (2015), where it is referred to as “Path efficiency,” this metric is used to identify excessively long paths. Such higher path lengths could be caused by wide deviations from the shortest path as well as numerous goings and comings around the target, which are two examples of poor trajectory control.
4. *Maximum Overshoot Ratio (MOR)*: ratio of the longest distance between the starting point and a reached point, by the distance to the target. This longest distance corresponds to the length *OS*. MOR is derived from metrics assessing overshooting movements in Casiez et al. (2008) and Hahne et al. (2015). It aims at quantifying overshoot magnitude instead of counting target-leaving occurrences, in order to distinguish short goings and comings in the close vicinity of the target, from wide and fast overshoots passing through the target without stopping onto it.

These metrics provide a quantitative evaluation of the performance that can be achieved with the system in the context of the task, by addressing various dimensions of motor performance, among which movement time, accuracy, stability, and trajectory control. Given that these different aspects may not be strictly independent in the context of a target-reaching task (Tresilian, 2012), some of the proposed metrics may overlap, as well as present conflicting results. However, employing these multiple metrics allows the performance assessment to compare how well each control mode can perform regarding a single dimension, and reveal if different modes correspond to different performance tradeoffs between these aspects. Besides, by identifying where each mode has its advantages and drawbacks, this approach can provide valuable insight regarding applications for prosthetic devices.

The force transducer used as the command device is purely isometric, so we expected better results on these metrics for velocity controls, based on the conclusions of Zhai (1995) regarding compatibility between order of control and type of command device.

At the end of each series, a 16-item questionnaire was given to the subject. Its first ten items are drawn from the System Usability Scale [SUS (Brooke, 1996)], a scale that was developed as a tool to quickly assess the usability of digital interfaces on computer screens, focusing on aspects such as ease of use, apparent complexity and learnability. We adapted this scale to the experimental context, which deals with the control of a robotic arm instead of a digital interface, and then translated it in French (see Supplementary Material). Our adapted SUS employs the same Likert scale basis as the original SUS: sixteen statements are presented along with a scale ranging from 1, “Strongly disagree,” to 5, “Totally agree” and the subject assigns a score to each statement, indicating to what extent he/she is in agreement with what is stated.

A global usability score out of 100 points is calculated based on the individual scores on the first ten items, according to the method provided by the original SUS. This score provides a general measure of the system’s usability based on the subjective evaluations from a population of users. The last six items are original items and address more specifically tiredness, frustration, and appearance of the robot’s movements, and are not involved in the calculation of the global SUS score. The end of the questionnaire also gives the subjects an opportunity to make free comments about the experiment, the system or the way it works.

Subjects were asked to complete the questionnaire items in the order of appearance. At the end of the experiment, after the questionnaire of the third series has been completed, the subject was asked to rank the three modes by order of preference, without the opportunity to refer to the previously completed questionnaires.

We performed Kruskal–Wallis tests on the results from the five quantitative measurements, the adapted SUS scores and the six supplementary questions scores, to detect significant differences between modes. In all the cases where these tests indicated the existence of such differences, *post hoc* Wilcoxon tests were performed to identify the pairs of modes presenting significant differences. All relevant statistical values from these tests can be found in Tables 1 and 2.

**Table 1:** Numerical outputs from Kruskal–Wallis tests.

	Performance metrics						
	AT	VT	PS	MOR	Success rate		
Degrees of freedom	2	2	2	2	2		
*χ*^2^ value	3.815	142.0	245.1	96.95	65.39		
p-value	0.1485	1.466 × 10^−31^	5.959 × 10^−54^	8.851 × 10^−22^	6.308 × 10^−15^		

	**Questionnaire**						
	**SUS score**	**Q11**	**Q12**	**Q13**	**Q14**	**Q15**	**Q16**

Degrees of freedom	2	2	2	2	2	2	2
*χ*^2^ value	12.51	5.102	10.56	0.8945	1.855	8.782	18.42
p-value	0.001923	0.07802	0.005085	0.6394	0.3956	0.01239	9.983 × 10^−15^

**Table 2:** Numerical outputs from Wilcoxon tests.

		Performance metrics			
		VT	PS	MOR	Success rate
Number of simultaneous tests		3	3	3	3
Bonferonni corrected p-value		0.0167	0.0167	0.0167	0.0167
Pos versus Vel	Z value	16,470	4,039	16,543	76.5
	p-value	5.160 ⋅ 10^−16^	5.254 ⋅ 10^−47^	6.989 ⋅ 10^−16^	4.892 ⋅ 10^−10^
Pos versus Vel2	Z value	12.317	5.041	16.965	79.5
	p-value	1.798 ⋅ 10^−24^	7.099 ⋅ 10^−44^	3.930 ⋅ 10^−15^	1.782 ⋅ 10^−10^
Vel versus Vel2	Z value	30.336	27.665	30.681	450
	p-value	0.2757	0.01461	0.3599	0.5930

		**Questionnaire**			
		**SUS score**	**Q12**	**Q15**	**Q16**

Number of simultaneous tests		3	3	3	3
Bonferonni corrected p-value		0.0167	0.0167	0.0167	0.0167
Pos versus Vel	Z value	12.0	9.0	7.0	6.0
	p-value	0.002254	0.005619	0.01059	0.003074
Pos versus Vel2	Z value	12.5	12.0	15.0	0.0
	p-value	0.004088	0.009558	0.05719	0.0008337
Vel versus Vel2	Z value	49.0	12.5	23.0	8.0
	p-value	0.8235	0.7921	0.3593	0.1317

## 3 Results

### 3.1 Qualitative Observations on Trajectories

**Figure 4:**
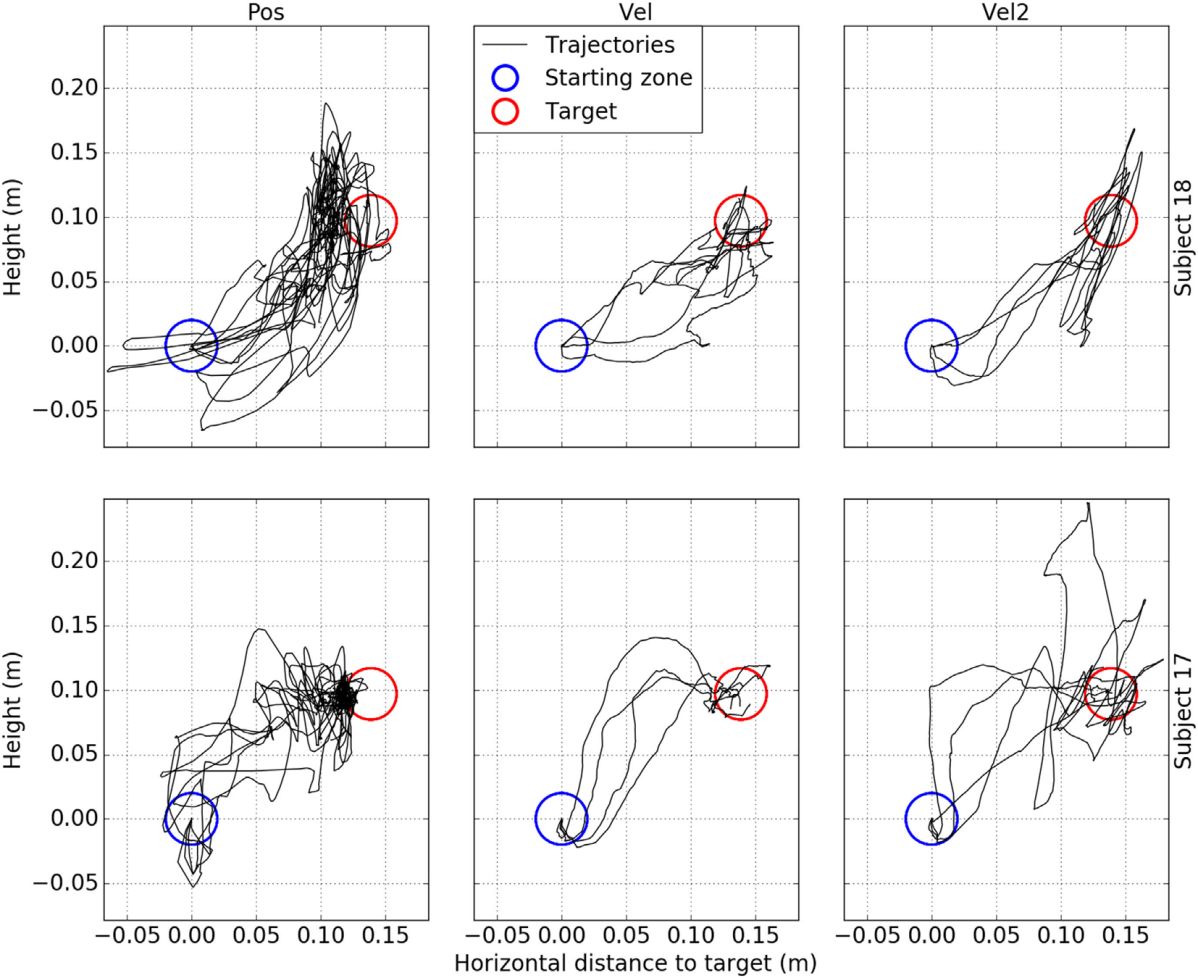
Examples of endpoint trajectories generated with each control mode. The data are taken from twenty-four trials performed by subjects #17 (bottom row) and #18 (top row). Each chart illustrates four trials performed by the same subject with the same mode and aiming at the same target (target B). Each column corresponds to one of the three control modes. The trajectories (in black), target (in red) and starting zone (in blue) are projected onto the vertical plane joining the starting point and the target center.

As a first step, qualitative study of the endpoint trajectories revealed notable differences in the way some subjects put the robot arm in motion in order to reach the targets. Figure 4 illustrates twenty-four trajectories performed by subjects 17 and 18 during all their trials requiring to reach target B. For better visualization, these 3-D trajectories are projected onto the vertical plane joining the starting point and the target center. In the illustrated cases, position control tended to produce unstable trajectories, containing many sharp direction changes as well as goings and comings, back and forth toward the target or widely oscillating around the shortest path, a straight line. Besides, we can note that these trajectories were longer than the ones produced by the other modes, but rarely overshoot the targets. Indeed, when the endpoint was inside the target zone, it mostly explored its nearest half.

On the other hand, trajectories produced with proportional velocity control appeared to be smoother and more regular towards the target, not too distant from the shortest path. Target leaving occurred rather less frequently than with Pos mode. The trajectories did not concentrate on the nearest part of the target zone. Quadratic velocity control tended to produce not-so-smooth trajectories, often close to a sequence of multiple segments, with some sharp turns. The hold-inside phase did not look as stable as with proportional velocity control, and the endpoint moved further from the center in target-leaving situations. These examples illustrate the fact that Vel2 mode tended to generate high speed movements more easily than Vel mode. Vel2 mode produced more overshoot and dragged the endpoint further, while Vel mode made the robot move slow enough to allow the user to correct the trajectory before it went too far in a wrong direction.

### 3.2 Quantitative Assessment of Performance Metrics

**Figure 5:**
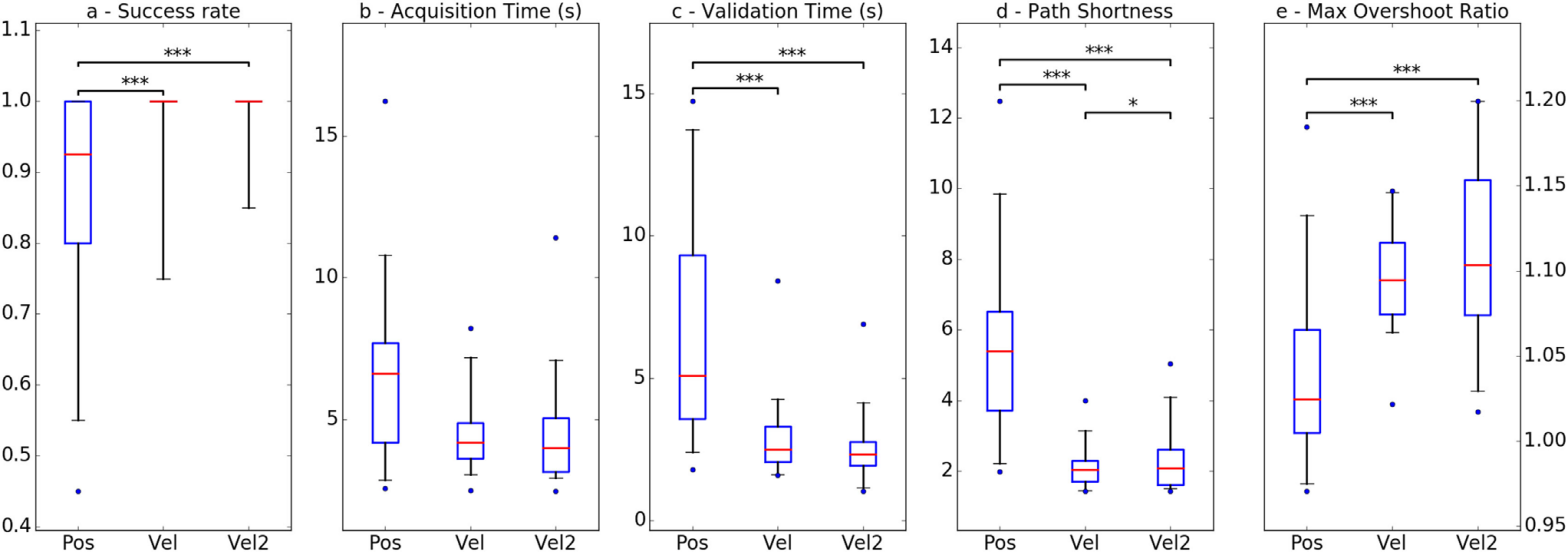
Evolution of performance between modes regarding the five quantitative metrics. Boxplots display the values’ distribution as follows: the box extends from lower to upper quartile values with a red line at the median, and the whiskers extend from the box to 5th and 95th percentile values. Flier points are considered outliers. Pairs of modes presenting significant differences are indicated with stars: *p < 0.05, **p < 0.01, ***p < 0.001. **(A)** Success rate, **(B)** acquisition time (s), **(C)** validation time, **(D)** path shortness, and **(E)** max overshoot ratio.

Overall, the subjects performed significantly less successfully with Pos mode than with any of the velocity modes (Figure 5A). More subjects achieved excellent scores (over 95% of successful trials) with Vel and Vel2 modes, although most of the subjects achieved good scores or better (over 80% of successful trials) with the three modes.

When it comes to movement time, no significant difference was found between modes regarding the time spent to reach the target zone (Figure 5B), but when they used Pos mode, it took subjects more time to successfully hold the endpoint within the target zone (Figure 5C). This indicates that placing the endpoint at a given position was not as easy with this mode as with the velocity control modes: subjects needed more time to accurately maintain it onto the target.

Path Shortness was the only performance metric to reveal significant differences for each pair of modes, Vel mode being the one allowing for the shortest path on average (Figure 5D). However, it is worth noting that the difference between the two velocity modes was much shorter than the difference between Pos mode and any of the velocity modes. As previously illustrated with the trajectory examples, paths produced in Pos mode tended to comprise bends and sinuosities, often distant from the ideal path toward the target, which made the resulting trajectories significantly longer.

Regarding the magnitude of maximum overshoot when trying to reach the target, significant differences were found only between Pos mode and each velocity control mode (Figure 5E). Pos mode appeared to produce shorter overshoot, probably due to the fact that the furthest positions reachable by the endpoint are directly related to the maximum force input a subject can produce. Besides, the metric’s variability was also quite high relatively to the difference in means between the three groups, revealing a wide range in the quality of the control achieved by the subjects with a given mode. Some of them proved to be able to drive the robot with enough accuracy to avoid bringing the endpoint much too far, whereas other produced wide, fast ballistic-like movements that unexpectedly and greatly overshot the target.

### 3.3 Usability Questionnaire Results

**Figure 6:**
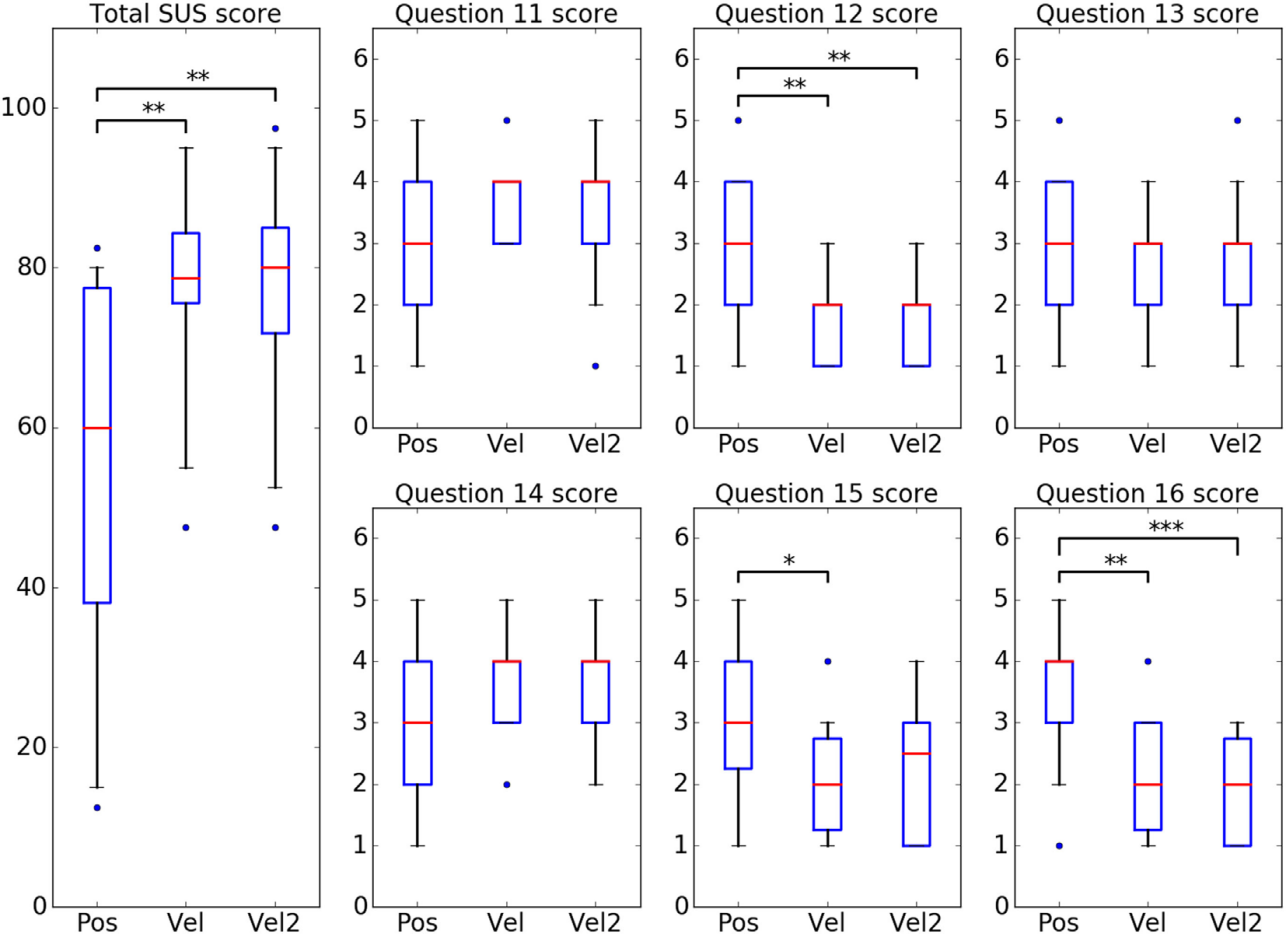
Questionnaire results on SUS and supplementary questions scores. Same boxplots as previously. Pairs of modes presenting significant differences are indicated with stars: *p < 0.05, **p < 0.01, ***p < 0.001.

Third, we performed Wilcoxon tests on the results from the questionnaire, divided in three groups corresponding to the three control modes. The global usability score results, calculated from the ten first items, presented significant differences between Pos and the two velocity control modes, whereas these two modes were not distinguishable regarding this global score (Figure 6, total SUS score). Even if, individually, most subjects felt notable changes from one velocity control to the other, the overall scores did not bring out any discernible variations between them according to this adapted SUS. However, the scale used in this work is not similar enough to the standard SUS to allow for comparison with scores from the original questionnaire regarding other systems, and the global score does not provide any information regarding the aspects where this advantage is the most distinct.

The results on the second part of the questionnaire were analyzed separately, item by item (Figure 6, questions 11–16). Wilcoxon tests were performed on the individual scores, divided in three groups in the same way as previously. Three questions about the robot’s movements, respectively, regarding conformity with the subject’s intentions (#11), human-likeness (#13) and timing according to the subject’s intentions (#14), did not present any significant difference between the three modes. This is consistent with the fact that the electro-mechanical setup, comprising robotic device, low-level motor operation, and inverse kinematics process, was common to all three experimental modalities. Indeed, the posture generation and the dynamic behavior of the robotic arm (motor responsiveness, inertia, etc.) remained the same during the experiments.

The question regarding the robot’s movements in terms of motion stability (#15) revealed significant differences between Pos and Vel modes. However, no significant difference was found between Vel2 mode and any of the two other modes for this question. On average, although the movements produced in Pos mode were not rated less consistent with what the subject intended to do, most of the subjects found the robot’s behavior in Pos mode more jerky and clumsy. Similar significant differences were also found for the two questions regarding the frustration (#12) and tiredness (#16) induced when using the system. Overall, Pos mode was rated more frustrating and tiresome than the two velocity control modes.

**Figure 7:**
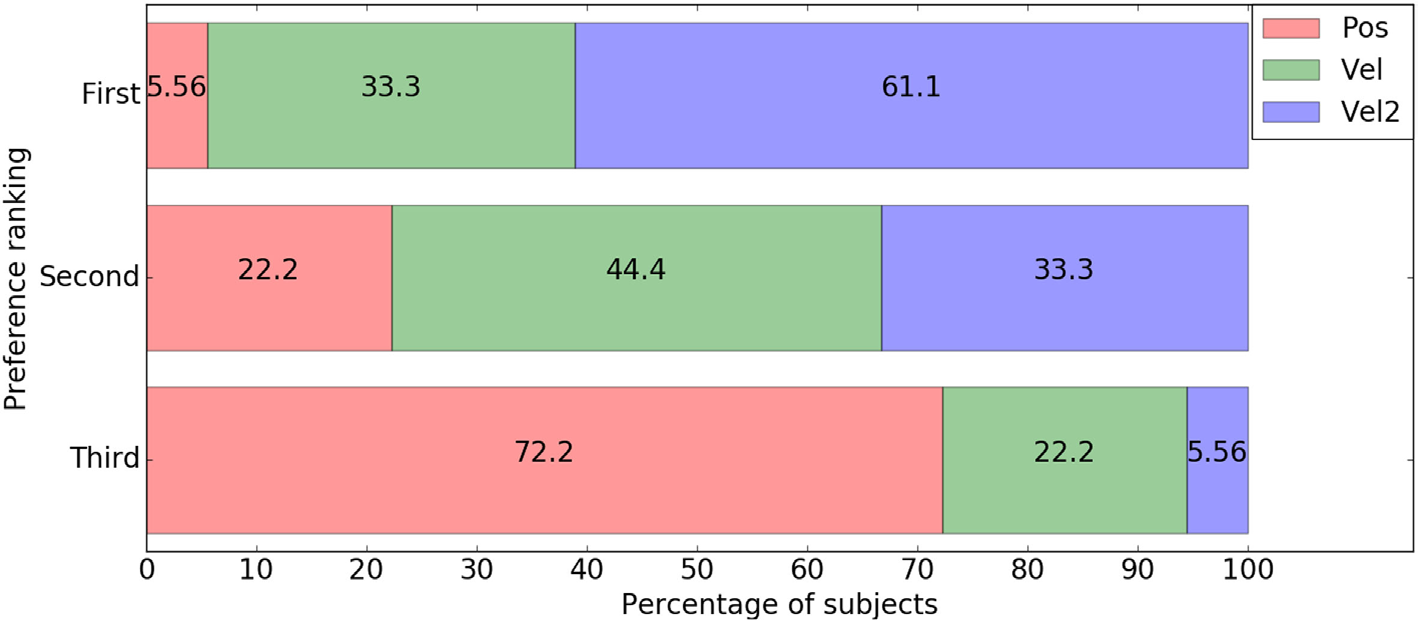
Distribution of mode preference rankings.

Regarding the preference orders provided by the subjects (Figure 7), we can see that Pos mode was the most negatively evaluated, with more than two-thirds of them ranking it last, and only one ranking it first. Similarly, Vel2 mode was the most positively evaluated, with more than half of the subjects ranking it first, and only one ranking it last. Globally, the preference difference between Vel and Vel2 is less distinct than between Vel and Pos, but still clear enough: two-thirds of the subjects ranked Vel2 mode better than Vel mode.

## 4 Discussion

### 4.1 Position Control versus Velocity Control

The results of our study suggest that, in the case of a force-driven robotic arm, velocity control allows for better performance than position control during a target-reaching task in the 3-D space. Overall, participants produced more successful and accurate motion when they used a velocity control mode to move the arm’s endpoint. Although no significant difference was found for Acquisition Time, implying that movements were not faster with one control mode over another, qualitative study of trajectories suggests that position control produced more unstable behaviors. Similarly, as shown by the results on Path Shortness, the trajectories in Pos mode seemed to grow excessively longer, owing to the endpoint oscillating around the target or the robot arm going back to its initial position when the subject releases its effort. It is probably due to the fact that, with Pos mode, placing the endpoint onto the target required the subject to produce a force vector closely matching with the ideal command vector in terms of both direction and magnitude, whereas a velocity control mode only required the force vector to be in the direction of the target.

Moreover, when it comes to fine control, the poorer maneuverability of Pos mode was illustrated by significant differences in Validation Time: subjects spent more time adjusting and holding the endpoint onto the target when using position control. This could be explained by the fact that maintaining the endpoint at a given position required the subject to hold a constant effort on the handle, while a velocity mode allowed the subjects to easily hold the endpoint still, just by releasing all effort on the handle. This specific behavior of Pos mode also made the robot move back toward its starting position as soon as the subject released or reduced the effort. Several subjects reported that they found this “spring” effect rather annoying, often reducing to nothing attempts at reaching the target when the subject cannot manage to hold the effort. Conversely, in velocity control, releasing the effort made the robot instantaneously cease its motion. Thus, the path already traveled was not lost, and the dwelling phase required much less, if any, effort production by the subject.

Regarding Maximum Overshoot Ratio results, overshoots proved to be significantly shorter for Pos mode than for any of the velocity control modes, but the latter did not seem to be severely penalized by their greater overshoots, given that their success rates are higher than with position control. The significant differences in Maximum Overshoot Ratio still illustrated key differences in behavior between the two orders of control. Indeed, with Pos mode, the further the endpoint went, the harder it became for the subject to make it move even further, as it required a bigger effort as input. Conversely, with velocity modes, the subject’s physical limits only restrained the endpoint’s movement speed and not its reachable positions, allowing for longer overshoot.

*Post hoc* analyses revealed that the global performance achieved with Pos mode was significantly lower than with Vel or Vel2 mode, according to four out of these five quantitative measurements. Besides, the only significant difference identified between Vel and Vel2 corresponded to Path Shortness, and the magnitude of this difference was considerably lower than that observed between Pos mode and the two velocity modes. These results suggest that in the context of this task, velocity control, whether it is proportional or quadratic, achieves better performances than position control. The fact that the two velocity modes did not clearly distinguish from one another might be due to the task itself, unfit to reveal differences between them.

Regarding questionnaire results, the significant differences that were found showed a clear advantage of velocity control from the user experience’s perspective. First of all, the global usability level evaluated by the adapted SUS was significantly lower for Pos mode, showing that overall, subjects had a better experience with a velocity control than with a position control during the tasks. Besides, several items from the second part of the questionnaire highlighted differences on specific aspects of the user experience. According to the subjects’ ratings, Pos mode felt more tiresome than any velocity control mode, obviously because it required the subject to apply more and more force on the handle until the endpoint reached the target, whereas a velocity control allowed to reach it by applying a constant and possibly smaller amount of force. Similarly, holding the endpoint in place required the subject to maintain an effort with Pos mode, while the same was done with a velocity control by simply releasing any effort on the handle. Pos mode was also found more frustrating than the two other modes, a result probably due to the behavior of this control mode when the subject had to produce enough force to reach the target. If the subject released the effort, the “spring” effect previously mentioned occurred, making the endpoint go back to the starting position. Finally, subjects rated Pos mode as more clumsy and unstable than the other modes. This is probably due to subjects having a harder time controlling accurately the endpoint while applying several Newtons of force during a few seconds, leading to the presence of short unwanted deviations in the trajectories.

These results from the questionnaire corroborate the results from performance metrics: position control was rated more negatively than velocity control on aspects related to user comfort and ease with the system. This is likely related to the quite distinct types of motion produced through position or velocity control: the former tended to generate fast movements, sudden or even brutal at times, whereas the latter tended to generate more moderately paced and smoother movements, that might have been easier to anticipate for the subject. This also highlighted the fact that employing usability assessment tools is relevant for evaluating control modes, as such tools complement performance evaluation by possibly confirming or contradicting its results. By addressing user experience, they can participate in carrying out a more thorough validation of the control modes, especially when it comes to user acceptability, which remains a key aspect in prosthesis design.

Our results in favor of velocity control over position control are congruent with the conclusions of Zhai (1995), stating that an isometric command device should be associated with velocity control for best compatibility. In the field of neural prostheses, Kim et al. (2008) also reported better performance in 2-D cursor control when decoding velocity than when decoding position from motor cortex activity with a Kalman Filter. On the other hand, several works report opposite results, in favor of a position control over a velocity control. Findings from Teather and MacKenzie (2014) remain in accordance with the conclusion of Zhai (1995) on compatibility between order of control and type of controller: position control is found to perform better than velocity control for driving a cursor with a 2-D tilt-sensor, which is an isotonic controller. However, Kim et al. (1987), addressing a pick-and-place task with a virtual telemanipulator, concluded that, when the work space is small or comparable to the human operators control space, position control performance is better than rate (velocity) control regardless of the type (isotonic or isometric) of joystick employed as the command device. This contradicts our results, which were similarly obtained with an isometric controller driving the robotic arm operating within a quite small workspace: the targets are located 19-cm far from the starting position, whereas the robotic arm is 49-cm long and the subject is seated only 1-m far from the target setup.

### 4.2 Linear versus Quadratic Transfer Function for Velocity Control

We introduced the quadratic transfer function to design a velocity control mode that would provide a better compromise between accuracy at low force input and fast movement at medium force input, so we expected better performance for Vel2 than for Vel mode. However, results from both quantitative measurements and usability questionnaire brought out only very few aspects on which these two modes distinguished from one another. The only metric to present a significant difference between them was Path Shortness, and mean values for each mode showed that this difference was not so pronounced (Δ = 0.251 for Vel versus Vel2) compared to the other significant differences from this metric (Δ > 3.28 for Pos versus Vel and Pos versus Vel2). Besides, in terms of performance analysis, a difference regarding Path Shortness does not represent an indication as strong as a difference regarding success rate or movement time. Even though we can conclude that trajectories performed with Vel2 mode tended to be a bit longer than with Vel mode, success rate and movement time did not appear to be significantly affected. Even when it comes to usability, despite the longer trajectories, Vel2 mode was not rated as more tiresome or less comfortable.

This lack of significant differences indicates that Vel and Vel2 modes were closely similar in terms of performance and usability, meaning that the advantage we expected from a quadratic function did not appear during the experiment. We can suppose that the experimental protocol, and especially the task, were unfit to highlight differences between the two velocity modes. Indeed, both presented very high success rates, suggesting, overall, that the task was not difficult enough to bring out how one mode could perform better than the other. We believe that designing a more challenging task, whether it is achieved by changing scalar gain values, target locations or even task nature, would help distinguish the two modes based on their respective results. For example, an alternative to the standard center-out target-reaching task is the pinball task, described by Gilja et al. (2012) and consisting in a single, continuous trial during which the subject drives the endpoint from one target to another. By requiring the user to produce movements with greater amplitude, in a wider variety of directions or toward unpredictable target locations, a pinball task would be more likely to reveal clearer differences between the two velocity modes and provide results in favor of one mode over another.

However, despite both velocity modes obtaining very close results, user preference is in favor of quadratic velocity control. Indeed, twelve out of eighteen subjects ranked Vel2 mode higher than Vel mode in their preference order, and among them eleven ranked Vel2 as the best mode. In this way, subjective user evaluation revealed a difference that could not be identified according to the other qualitative and quantitative elements we employed to assess the different modes. These results somehow suggest that these assessment tools and metrics may not be fine enough in the context of this experiment, and also highlight the fact that asking subjects to rank the tested modes by general preference is a relevant element for comparing them.

### 4.3 Influence of the Experimental Setup

#### 4.3.1 Pointing Device

The congruency between our findings and the conclusions of Zhai (1995) tends to indicate that our conclusions on velocity control outperforming position control are not specific to the robot arm employed here as the pointing device. Indeed, similar results were obtained in various contexts such as 3-D virtual reality space (Zhai, 1995) and 2-D cursor control on a computer screen (Kim et al., 2008), supporting the hypothesis that compatibility between order of control and nature of the controller is independent of the nature of the pointing device.

#### 4.3.2 Controlling Device

This congruency also highlights the fact that our results seem to be quite specific to the isometric nature of the used sensor, but not necessarily to the direct measurement of static linear efforts. Indeed, we would expect different results with a 3-D isotonic device such as a Wii Remote™, but comparable results with another isometric 3-DoF device, such as the "EGG" (Elastic General-purpose Grip) controlling device introduced by Zhai (1995). This device takes the shape of a palm-sized ball held at the center of a rigid square frame by elastic springs, and sensors placed inside the ball are used to compute its displacement relatively to its resting position, at the center of the frame. Contrary to the force transducer used in our work, this controller does not perform effort measurements and is not static, as the ball can slightly move within the frame. However, the springs pulling the ball back to the center make this device an isometric controller, expected to allow for better performance when associated with velocity control.

Zhai (1995) carried out an experimental comparison of the performance achieved by velocity control with this elastic controller (opposing resistance, short movement range) versus a static controller (stiff, no movement range), the Spaceball^TM^, another isometric 6-DoF controller. This experiment revealed no substantial difference in performance between elastic and static controllers, as long as they were isometric, supporting our hypothesis that our conclusions on performance are not specific to the static nature of the used sensor. Such alternative controllers could be employed in future experiments, allowing to investigate the extent to which our results are specific to the use of a static force transducer.

#### 4.3.3 Task Format and Parameters

Our experimental setup being physical rather than virtual, the starting position and target placement is heavily constrained by the boundaries of the robot arm’s reachable space, as well as the volume occupied by each target assembly. Although the targets were placed so that a wide portion of this space is covered during an experimental series, the number of targets and their distance to the starting position remain limited. Provided that the pointing device is still able to reach them, placing the targets further from the starting position would make the task more difficult overall. Whereas the former velocity control modes would still allow the subject to perform the task successfully, such a modification would require the use of a higher gain for Pos mode, in order to allow the endpoint to reach a further target without requiring an excessively high amount of force produced by the subject. This increase in sensitivity would probably make the endpoint harder to hold in place and result in higher Validation Times. A longer distance to target may also reveal clearer differences between control modes in terms of Acquisition Time, especially between Vel and Vel2 modes. Indeed, Vel2 mode is able to produce faster movements than Vel mode with medium force input, a property that should represent a major advantage when attempting to reach a distant target.

On the other hand, the hold-inside duration required to successfully perform the task can be chosen independently from the experimental apparatus. Given that the hold-inside phase is found to be more difficult with Pos mode, we would expect higher differences in Validation Time for a longer hold-inside duration. In terms of force input, Pos mode requires the subject to maintain the effort on the handle in order to hold the endpoint onto the target, whereas Vel and Vel2 modes hold the endpoint still when no force is applied on the sensor. Consequently, extending the hold-inside phase duration should make the task more tiresome in Pos mode, while in Vel or Vel2 mode the subject needs to exert little to no effort during this phase. Besides, regarding the post-experiment questionnaire results, the ratings on questions #12 about frustration and #16 about tiredness would probably remain significantly different between Pos mode and each velocity mode.

The target-reaching task employed in our experimental protocol follows a center-out format, where the operational space comprises a central position around which the targets are placed and each trial corresponds to a single target. This format is widely used in the field of neuroprostheses (Kim et al., 2008; de Rugy et al., 2012b; Gilja et al., 2012; Sussillo et al., 2012; Golub et al., 2014; Hahne et al., 2015) to assess the motor performance of a neuroprosthetic control technique. However, alternative formats could be employed in order to further investigate the performance and usability of our system. For example, a center-out-and-back format, described in Gilja et al. (2012) and similar to the protocol employed in Hahne et al. (2015), requires the endpoint to reach a target then go back to the starting position. This would introduce a second movement phase in the task and help study how the different modes allow the system to perform direction changes. Besides, the “spring” effect particular to Pos mode would become a decisive advantage in the context of this task, as releasing all efforts on the handle would immediately bring the endpoint back toward the starting position. Thus, we would expect with this mode a much higher movement time when attempting to reach the target than when going back to the center. Regarding velocity control modes, which would not benefit from this effect, we would expect results in Acquisition Time from center to target and vice versa to be comparable to what is achieved on a center-out format.

Another alternative task format is the aforementioned pinball task, comprising multiple movement phases during which the subject must drive the endpoint from one target to another. Contrary to a center-out format, the distance to travel during each movement phase may not be constant, making it impossible to compare Acquisition Time from one target to the next. However, this metric could be normalized by the distance to travel, as well as complemented by a measurement of the overall completion time. Due to the variations in travel distance, this task format may bring out differences in Acquisition Time between Vel and Vel2 modes, since the latter allows the endpoint to move at higher speeds. Moreover, given that a trial would span the whole target set without any pause, tiredness would probably build up as the trial goes on, possibly leading to critical control issues with Pos mode, which is known to be more tiresome. Indeed, with this mode, subjects would be more likely to experience fatigue after going through several targets, causing the endpoint control to become more difficult.

In summary, we hypothesize that our results regarding velocity control outperforming position control are not specific to the task featured in our protocol, whereas distinguishing Vel and Vel2 modes in terms of performance will be possible by investigating different experimental configurations and tasks.

### 4.4 Limitations and Future Work

#### 4.4.1 Choice of Gain Values

Our study used constant scalar gain values that were rather arbitrarily chosen by the experimenters after having conducted an informal pilot testing. Contrary to Kim et al. (1987), who chose the gain settings from rigorous pilot testing conducted with the same subject population prior to the main experiment, this work did not compare different control modes set with gain values considered as “ideal.” No such argument was provided in the present study to justify the choice of scalar gain values, and this lack of exhaustiveness brings out the need to address more thoroughly this aspect in future works.

Besides, the gain values used here were common to all subjects, regardless of their physical abilities, which appeared as potentially important during tasks with Pos mode. Indeed, producing enough effort on the handle so that the endpoint reached the target seemed to be an issue itself for a few subjects, independently of their ability to make the endpoint move in the right direction or hold it in place. Employing user-specific gain values is a typical solution to this kind of issue, but raises the need to elaborate relevant methods to determine these gain values for each user. For instance, a method, inspired by the protocol from de Rugy et al. (2012b), is to determine gain values as a function of the maximum effort that the user is able to produce on the handle, so that a nominal displacement or speed level can always be reached by any user. This would require the addition of a user-specific calibration phase to the current protocol.

Furthermore, as mentioned by Teather and MacKenzie (2014) and addressed by Casiez et al. (2008) with pointer acceleration, employing dynamic gains rather than gains remaining constant throughout a task is an alternative worth exploring. For example, based on the assumption that fast device movement implies that a great distance must be covered, some techniques used for mouse-driven cursor control dynamically amplify the CD (Cursor-Display) gain when the mouse velocity rises, allowing for quicker cursor movement than with a constant CD gain. Such techniques do not apply to purely isometric controllers such as the force transducer employed in the present work if they rely on device velocity, but could be adapted in future works to modify gain values based on the first derivative of the force input.

#### 4.4.2 Perspectives on Complementary Performance and Usability Evaluation

Regarding robustness, our study did not formally address how the different control modes react to disturbances, such as noise affecting the command vector. This aspect plays a notable role in the control of a myoelectric prosthesis, whose input command signals can be subject to unwanted external variations, whether they are caused by the subject’s physiological activity itself (e.g., involuntary contractions, muscular fatigue), or by the measuring conditions (e.g., sensor positioning, sweat under the electrodes). While some of these disturbances could be filtered through signal processing or recalibration, the command signals representing movement intention may still be affected. As for the system employed in this work, disturbances on the command vector would produce different effects depending on the control mode, especially when the command vector is intended to remain as a null vector (no effort applied on the transducer). Indeed, in this situation, Pos mode would react to noise by making the endpoint shake and move around the starting position, within a space directly bound by the noise magnitude. On the other hand, Vel and Vel2 modes would make the endpoint drift away from the starting position, potentially until it gets to the reachable space’s limits, if the system keeps running without being driven for a long time. Following this example, studying how each mode behaves when confronted to noisy command signals represents a major aspect to address in future works. Indeed, although velocity control modes are found here to perform better than Pos mode, such works will provide complementary results for an overall performance assessment, possibly revealing situations where Pos mode outperforms velocity control modes. Moreover, addressing robustness will contribute to their comparison regarding dimensions of usability that are more closely related to human factor aspects.

Concerning performance and usability evaluation, a key perspective lies in refining the assessment tools and methods used to compare control modes, in order to address more accurately the factors influencing and determining user preference. Indeed, the metrics and criteria employed in the present work revealed only one slight difference between proportional and quadratic velocity controls, a result quite insufficient to explain subjects’ global preference being in favor of the latter over the former. Regarding these findings, an aspect worth exploring is whether this subjective preference is rooted in actual advantages of a transfer function over the other in terms of quality of control or in factors more closely related to user experience.

#### 4.4.3 Perspectives on Prosthetic Application

Even though a force-driven device does not seem suitable for prosthetic application, the current system could be integrated into an arm prosthesis with the help of an EMG-based interface converting myoelectric signals into a force command vector. Such an interface can rely on a biomechanical model of the upper limb (Manal et al., 2002) to simulate muscular efforts corresponding to a given set of EMG measurements, and compute the resulting limb forces. Alternatively, de Rugy et al. (2012b) proposed a user-specific method to reconstruct forces produced by the wrist from EMG measurements on upper arm muscles, using linear regression techniques. These approaches could be adapted to decode a 3-D force vector from myoelectric signals measured on the remaining muscles of a subject suffering from upper-limb disability.

However, the implementation of such techniques in the context of a disabled limb introduces new technical issues to be solved. First of all, regression- or reconstruction-based techniques rely on measurements and analysis of native limb motion, which obviously cannot be fully performed with a disabled limb. To allow disabled subjects to take part in the process, a solution is to perform the regression against intended movements instead of actual movements (Hahne et al., 2015). On the other hand, the choice of muscles from which to measure myoelectric signals will necessarily depend on the nature of the upper-limb disability (e.g., transradial, transhumeral) and affect the control of the robotic arm. This issue is one of the main perspectives to address in future works aiming at prosthetic application.

Furthermore, it is clear that performance levels achieved with the current system do not compare with those achieved by native arm movements, whether it be in terms of movement time (typically 5 to 15 s versus less than 1 s) or dexterity (sharp-edged and erratic trajectories versus smooth and gently curved paths). This points out that major progress still has to be done on our system before considering any application for arm prostheses, whose context of use is far more demanding and diverse than the task featured in our experimental protocol. Regarding trajectory control, filtering techniques could be integrated to the processing chain, either on input command signals or on output kinematics, in order to produce smoother endpoint motion as well as enhance stability and noise tolerance. On the other hand, improving movement speed is a quite challenging issue, given that allowing for faster movements by increasing gain values will probably result in poorer fine control at low speed. We believe that employing non-linear transfer functions can play a decisive role in the resolution of this issue by introducing versatility and flexibility in the control process.

## 5 Conclusion

Our study investigated a key aspect of robotic prosthesis control: the choice of the method converting physiological input command into prosthesis motion. Using a force transducer as the command device driving a robotic arm’s motion, we compared position and velocity control in the context of a target-reaching task, and demonstrated that the latter performed better in these conditions. In addition to performance metrics, widely used in the literature on mobile device control, we introduced usability metrics among the evaluation criteria employed to assess the different control modes. Through a post-experiment questionnaire given to subjects, we observed congruency between results from performance metrics and from usability assessment: velocity control proved to perform significantly better than position control in the context of this task. These findings confirm that user experience quality tends to reflect the relative levels of performance allowed by the different control modes. However, the study of user preference also showed that quadratic velocity control was globally favored by subjects over proportional velocity control, while results from other metrics and measurements did not reveal such a clear difference between the two velocity modes. This supports our assertion that, in the field of mobile device control and especially robotic prostheses, subjective user experience and appreciation go beyond objective performance and quality of control, pointing out the need to integrate usability assessment tools within the evaluation process of such systems.

## Ethics Statement

This study was carried out in accordance with the recommendations of the local ethics committee (CPP Sud-Ouest et Outre Mer III, Ref DC2014/16), with written informed consent from all subjects. All subjects gave written informed consent in accordance with the Declaration of Helsinki. The protocol was approved by the local ethics commitee.

## Author Contributions

SM designed, conducted, and analyzed the experiment and interpreted the results, under the supervision of P-YO and AR and with inputs from DC and FP. P-YO designed the robotic platform. SM wrote the paper with inputs from all other authors. All authors approved the final version.

## Conflict of Interest Statement

The authors declare that the research was conducted in the absence of any commercial or financial relationships that could be construed as a potential conflict of interest.
