# Supplementary material for: Performance and Usability of Various Robotic Arm Control Modes from Human Force Signals
Source: Front Neurorobot. 2017 Oct 25;11:55. doi: 10.3389/fnbot.2017.00055 (PMC5660981; doi:10.3389/fnbot.2017.00055)
Supplement: Supplementary file 1 [file image_1.pdf]

Subject   Date :

Modality

Evaluate each statement by assigning a score on the scale, indicating to what extent you agree with them.

		Strongly disagree				Strongly agree
		**1**	**2**	**3**	**4**	**5**
**1**	I think that I would like to use the system frequently					
**2**	I found the system unnecessarily complex					
**3**	I thought the system was easy to use					
**4**	I think that I would need the support of a technical person to be able to use this system					
**5**	I found the various functions in this system were well integrated					
**6**	I thought there was too much inconstency in this system					
**7**	I would imagine that most people would learn to use this system very quickly					
**8**	I found the system very awkward to use					
**9**	I felt very confident using the system					
**10**	I needed to learn a lot of things before I could get going with this system					

Turn please

		Strongly disagree				Strongly agree
		**1**	**2**	**3**	**4**	**5**
**11**	I thought the robot's movements were consistent with my intentions, compliant with what I wanted to do.					
**12**	I thought the system is annoying, frustrating when using it					
**13**	I found the robot's movements weird, unnatural, not human-like					
**14**	I thought the robot's movements happened in right time compared to my intentions					
**15**	I found the robot's movements jerky, unstable, clumsy					
**16**	I found this system tiresome to use					

**17**	Do you have comments or observations regarding the experiment you just participated in?
