# Supplementary material for: Performance and Usability of Various Robotic Arm Control Modes from Human Force Signals
Source: Front Neurorobot. 2017 Oct 25;11:55. doi: 10.3389/fnbot.2017.00055 (PMC5660981; doi:10.3389/fnbot.2017.00055)
Supplement: Supplementary file 2 [file image_2.pdf]

Sujet Date :

Modalité

Évaluez chacune de ces affirmations en indiquant à l'aide de l'échelle à quel point vous êtes en accord avec elles.

		Pas du tout d'accord				Tout à fait d'accord
		**1**	**2**	**3**	**4**	**5**
**1**	Je pense que j'aimerais utiliser ce système fréquemment pour piloter un robot					
**2**	J'ai trouvé le système inutilement complexe					
**3**	J'ai trouvé le système facile à utiliser					
**4**	Je pense que j'aurais besoin de l'aide d'une personne expérimentée pour être capable d'utiliser ce système					
**5**	J'ai trouvé la fonction du système robotique bien intégrée dans le dispositif					
**6**	J'ai trouvé qu'il y avait trop d'incohérences dans ce système					
**7**	J'imagine que la plupart des personnes apprendraient vite à se servir de ce système					
**8**	J'ai trouvé que le système était peu maniable à utiliser					
**9**	Je me sens en confiance lors de l'utilisation de ce système					
**10**	J'ai eu besoin d'apprendre beaucoup de choses avant d'utiliser le système					

Tournez S.V.P

		Pas du tout d'accord				Tout à fait d'accord
		1	2	3	4	5
11	J'ai trouvé les mouvements du robot cohérents avec mes intentions, en accord avec ce que je voulais faire					
12	J'ai trouvé ce système contrariant, frustrant dans son utilisation					
13	J'ai trouvé les mouvements du robot étranges, non naturels, peu humains					
14	J'ai trouvé que les mouvements du robot avaient lieu au bon moment, à temps, par rapport à mes intentions					
15	J'ai trouvé les mouvements du robot saccadés, instables, maladroits					
16	J'ai trouvé ce système fatiguant à utiliser					

17	Avez vous des remarques, des observations concernant l'expérience que vous venez de réaliser ?
